# Loss of *Gnas* Imprinting Differentially Affects REM/NREM Sleep and Cognition in Mice

**DOI:** 10.1371/journal.pgen.1002706

**Published:** 2012-05-10

**Authors:** Glenda Lassi, Simon T. Ball, Silvia Maggi, Giovanni Colonna, Thierry Nieus, Cheryl Cero, Alessandro Bartolomucci, Jo Peters, Valter Tucci

**Affiliations:** 1 Department of Neuroscience and Brain Technologies, Istituto Italiano di Tecnologia, Genova, Italy; 2 Medical Research Council Mammalian Genetics Unit, Harwell, United Kingdom; 3 Department of Integrative Biology and Physiology, University of Minnesota, Minneapolis, Minnesota, United States of America; University of Cambridge, United Kingdom

## Abstract

It has been suggested that imprinted genes are important in the regulation of sleep. However, the fundamental question of whether genomic imprinting has a role in sleep has remained elusive up to now. In this work we show that REM and NREM sleep states are differentially modulated by the maternally expressed imprinted gene *Gnas*. In particular, in mice with loss of imprinting of *Gnas*, NREM and complex cognitive processes are enhanced while REM and REM–linked behaviors are inhibited. This is the first demonstration that a specific overexpression of an imprinted gene affects sleep states and related complex behavioral traits. Furthermore, in parallel to the *Gnas* overexpression, we have observed an overexpression of *Ucp1* in interscapular brown adipose tissue (BAT) and a significant increase in thermoregulation that may account for the REM/NREM sleep phenotypes. We conclude that there must be significant evolutionary advantages in the monoallelic expression of *Gnas* for REM sleep and for the consolidation of REM–dependent memories. Conversely, biallelic expression of *Gnas* reinforces slow wave activity in NREM sleep, and this results in a reduction of uncertainty in temporal decision-making processes.

## Author Summary

REM and NREM sleep are two distinct stages of the sleeping brain that are involved in the modulation of metabolic, physiological, and cognitive processes. Clinical evidence suggests that epigenetic mechanisms, such as genomic imprinting, play a role in sleep. Here we show that REM and NREM brain states are differentially modulated by the maternally imprinted gene *Gnas*. In particular, a mutation resulting in loss of imprinted expression of *Gnas* enhances NREM–dependent physiologic and cognitive functions while repressing REM and REM-linked functions. This is the first experimental demonstration of a specific effect of genomic imprinting on sleep states and their associated effects on cognition.

## Introduction

Mammalian evolution from the reptile lineage involved important changes in gene regulation and sleep. Many genetic mechanisms play important roles in various electrophysiological and behavioral traits that set the three major states of mammalian life: Wakefulness, Rapid Eye Movement (REM) and Non-REM (NREM) sleep [1], [2]. However, here we focus on a particular gene regulation, namely genomic imprinting. Genomic imprinting is an epigenetic mechanism that results in allele-specific expression of some genes according to parental origin and, in vertebrates, is unique to mammals.

Clinical observations of neurodevelopmental disorders of sleep suggest a role of genomic imprinting on various measures of sleep [3], [4]. For example, Prader-Willi syndrome (PWS) and Angelman syndrome (AS), both neurodevelopmental syndromes, exhibit opposing imprinting profiles and opposing sleep phenotypes. PWS is associated with maternal duplications/paternal deletions of alleles on chromosome 15q11–13 and is characterized by temperature control abnormalities and excessive sleepiness as well as REM sleep abnormalities [5]–[8]. Conversely, AS is associated with paternal duplications/maternal deletions on chromosome 15q11–13 and is characterized by severe mental retardation and reductions in sleep. The *UBE3A* gene, that resides in the PWS/AS imprinting region, has been associated to sleep abnormalities. *Ube3a* deficient mice are characterized by reduced NREM sleep, deteriorated REM sleep, and an increased frequency of waking during the dark–light transition [9].

Interestingly, the serotonin (5-HT) 2A receptors, which mediate aminergic inhibition of REM-on cells in the parabrachialis lateralis region [10], are primarily expressed from maternal alleles [11], [12]. The above reviewed findings support the idea that epigenetic regulatory mechanisms, such as genomic imprinting, influence sleep-associated mechanisms.

REM and NREM sleep underlie important metabolic, physiological and cognitive processes. REM sleep influences early postnatal developmental behaviors; it facilitates the acquisition of resources from the mother (i.e. by means of suckling behavior) and it promotes the release of hormones such as prolactin and oxytocin which are pivotal in the development of attachment behavior [4]. From an evolutionary and behavioral perspective, REM sleep has been associated with adult reproductive success [4]. NREM sleep is associated with more stable metabolic and autonomic responses compared to REM sleep. Furthermore, the presence of REM-like and NREM-like states has been associated with nutritive behaviors in offspring and mother, respectively [4].

Recent advances in functional studies of sleep strongly suggest that memory consolidation benefits from a slow (<1 Hz) highly synchronized cortical activity in NREM and from subcortical theta (5–9 Hz) rhythms in REM sleep [13]. Despite these advances in functional understanding of REM and NREM sleep states, genetic and epigenetic mechanisms of sleep-dependent plasticity and memory processing are not currently well understood.

Here we report, for the first time, experimental evidence for a role of an imprinted gene, *Gnas*, in the modulation of REM/NREM sleep physiology. *Gnas* encodes the stimulatory G-protein subunit G_s_α, which is involved in the generation of intracellular cyclic AMP and plays a crucial role in energy expenditure and metabolism by mediating sympathetic effects on many tissues [14]. It is biallelically expressed in most tissues including adult white adipose tissue [15] but it is predominantly maternally expressed and paternally repressed in a subset of tissues such as neonatal brown adipose tissue (BAT) [16], although it is not known if it shows imprinted expression in adult BAT. BAT produces heat by fatty acid oxidation and serves a pivotal thermoregulatory function within the organism [17]. Thanks to the rich presence of mitochondria and by means of the uncoupling protein-1 (UCP1), BAT is implicated in non-shivering thermogenesis [15], [18].

**Figure 1:**
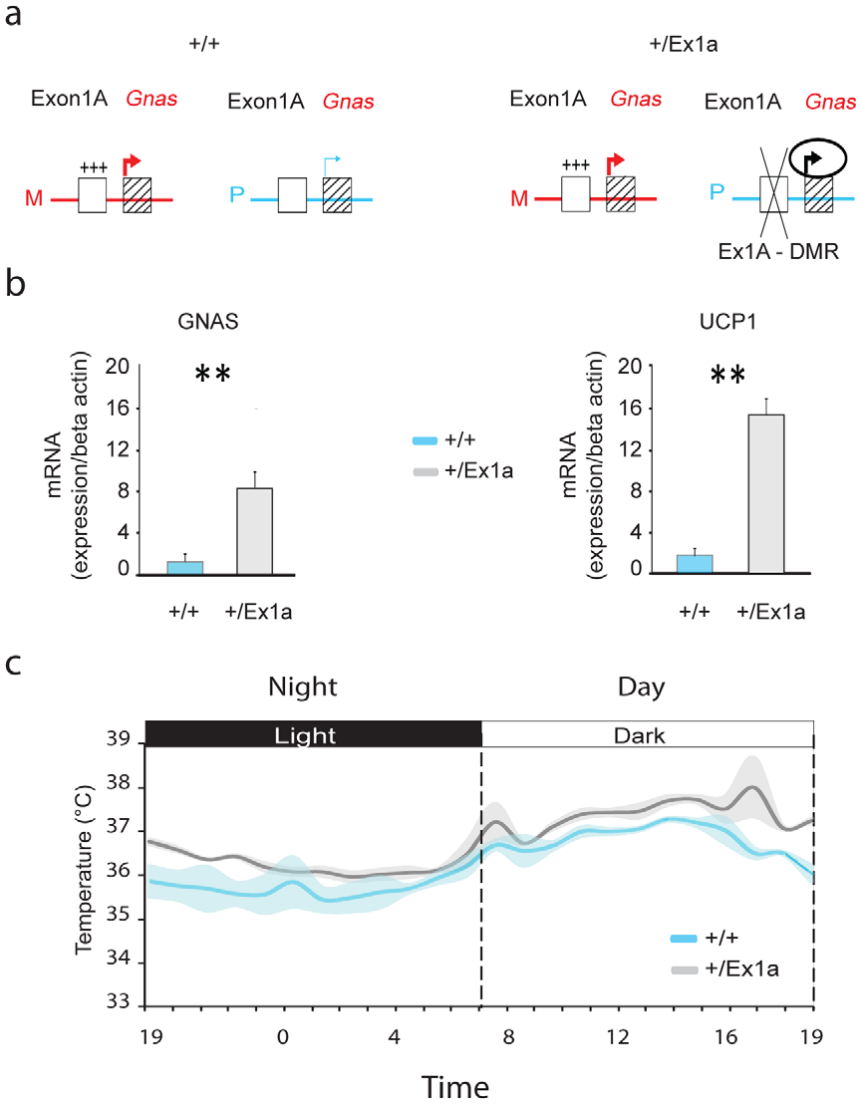
Exon 1A deletion: *Gnas* expression and body temperature. (a) Representation of *Gnas* expression when the *Exon1A-DMR* (Ex1A-DMR) is methylated on the maternal (M) allele in +/+ and +/*Ex1a* or deleted on the paternal (P) allele in +/*Ex1a* mice. (b) Expression levels, by q-PCR measurements, in BAT tissue of *Gnas* (left panel) and *Ucp1* (right panel) mRNA in *+/+* versus mutant *+/Ex1a* mice. (c) Grand-averages and shadowed ± s.e.m. of body temperature over a 24-hour period for +/+ and +/*Ex1a* mice. Statistics are reported as following: *t*-test; ** = *P*<0.01.

Imprinted expression of *Gnas* is controlled by a cis-acting differentially methylated region (DMR): the *Exon1A-DMR* [16]. Paternal transmission of a deletion of the *Exon1A-DMR* (*Gnas^tm1Jop^*, hereafter called *Ex1a*, see Figure 1a) causes derepression of the normally repressed paternal *Gnas* allele in imprinted tissues resulting in biallelic *Gnas* expression and loss of imprinting [16]. We show here, for the first time, that loss of imprinting of *Gnas* results in specific abnormalities in sleep, cognition and thermoregulation in adult mice.

## Results

On paternal inheritance of the deletion, we have observed that in *+/Ex1a* mice the level of *Gnas* mRNA expression is increased, compared to littermate controls, in adult BAT (Figure 1b) indicating a derepression on the paternal allele due to a loss of *Gnas* imprinting, as previously reported in newborn mice BAT [16]. Moreover, we show here that *Ucp1* mRNA in BAT is significantly higher in *+/Ex1a* mice compared to littermate controls (Figure 1b). As expected from the observation of increased *Ucp1* levels a significantly higher body temperature was found in mutant animals (Figure 1c). The major increase of temperature in *+/Ex1a* mice compared to littermate controls occurs at the end of the subjective day and at the beginning of the subjective night, when there is a strong urge to sleep (Figure 1c).

**Figure 2:**
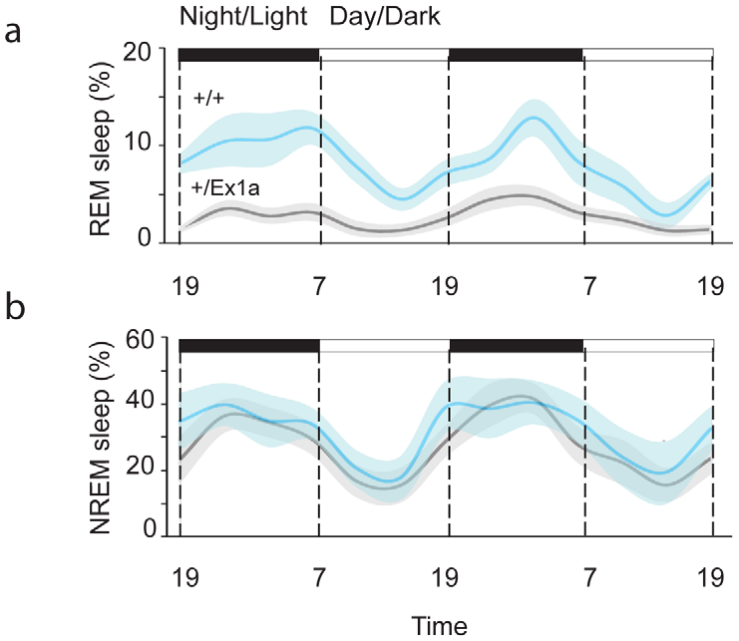
Sleep architecture in Exon 1A mice. Four-hour bins percentage and ± s.e.m., shadowed, of REM (a) and NREM (b) sleep for a 48-hour period.

**Figure 3:**
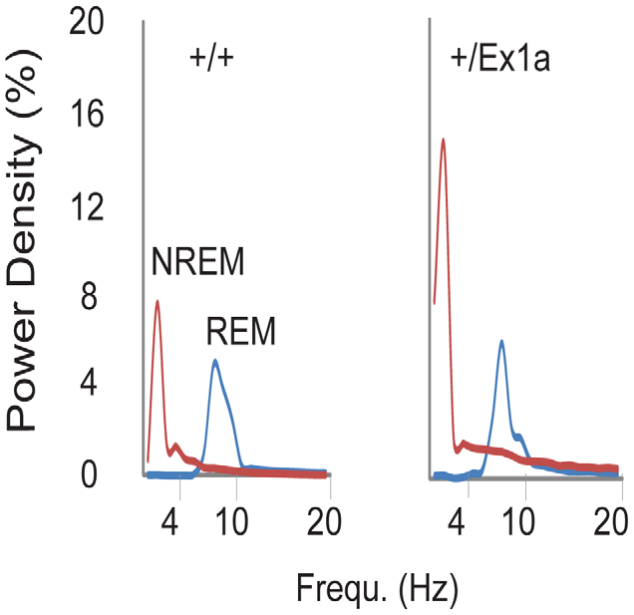
Representation of EEG frequencies in REM and NREM sleep. Here is represented the power density (see Methods) for all frequencies in all 4-seconds REM (blue line) and NREM (red line) sleep epochs during a 48-hour baseline.

In order to investigate the sleep-wake profile and the behavioral performance in these mice, we subjected adult *+/Ex1a* mice and *+/+* littermate controls to behavioral and electrophysiological investigation in the home-cage environment. Interestingly, the total REM sleep was significantly reduced in *+/Ex1a* mice compared to littermate controls (Figure 2a) while the total NREM was unaffected between the two groups (Figure 2b). However, at the electrophysiological level, the contribution (power density) of delta (1–4 Hz) frequencies, the main synchronized rhythm in NREM sleep, was higher in the *+/Ex1a* mice compared to *+/+* mice (Figure 3).

A 6-hour sleep deprivation protocol triggered, immediately after deprivation, a significantly increase (rebound) of REM in *+/Ex1a* mice, which testifies the need for a homeostatic recovery of REM, but not for NREM sleep, in mutants (Figure S1 and Figure S2). No differences in body temperature occurred during sleep deprivation and during the following recovery period between the two groups (Figure S2).

**Figure 4:**
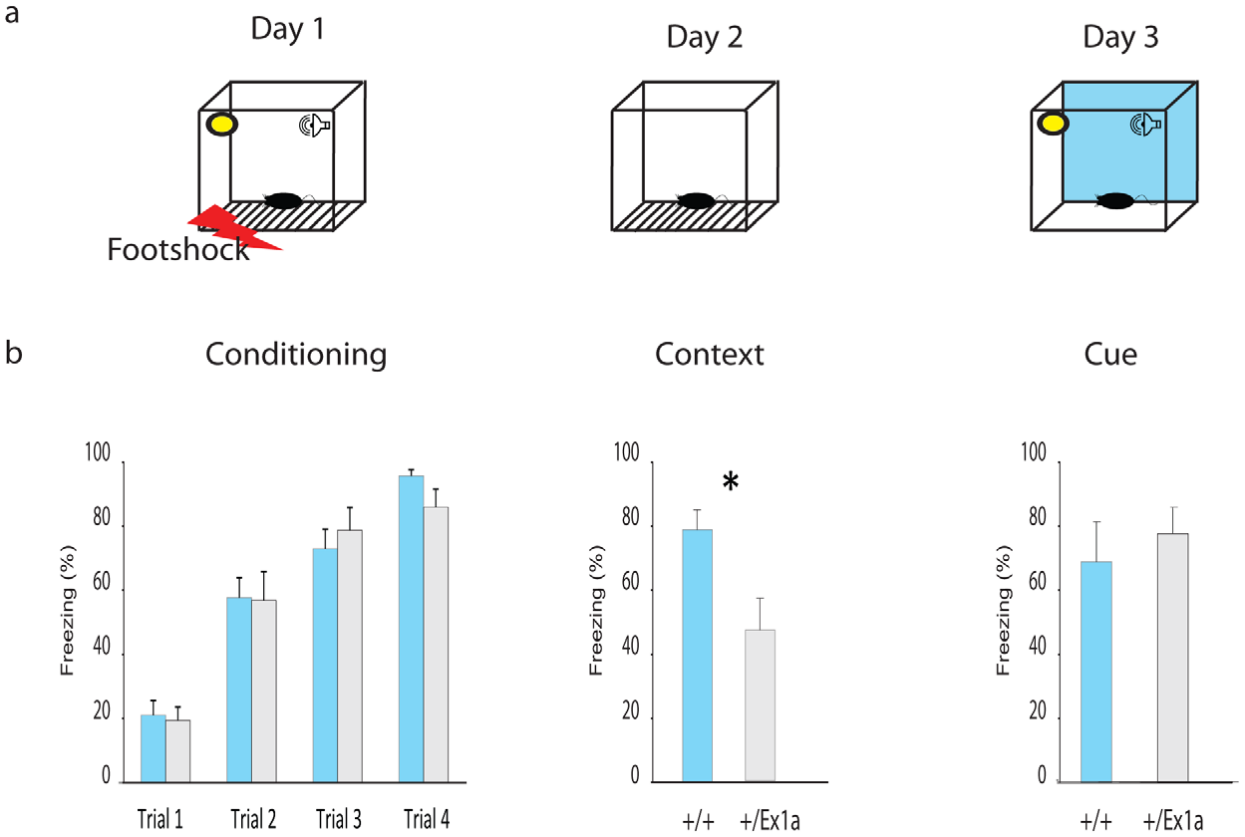
Fear conditioning performance. (a) Representation of the FC protocol over three days and three different experimental phases (Conditioning, Context and Cue). (b) Mean percentage values of freezing behavior ± s.e.m. are plotted for each experimental phase. Statistics are reported as following: *t*-test; * = *P*<0.05.

Thus, we extended our investigation into specific REM/NREM-dependent behavioral functions. We subjected mice to a classical memory task, the fear conditioning (FC) test, which affects REM sleep homeostasis [19]. After the first (conditioning) day of the FC protocol, we have observed an increase of REM sleep but not of NREM sleep in *+/+* mice (Figure S1a–S1b). The presence of REM and its theta density were significantly higher in *+/+* compared to *+/Ex1a* mice (Figure S1b). We suggest that this lack of REM increase in mutants is the causal mechanism explaining the reduced freezing behavior in *+/Ex1a* mice, compared to controls, that occurred when the animals were exposed to the same context (Figure 4) the following day. Indeed, the consolidation of fear responses has been previously associated with REM mechanisms that occur during sleep [19]. Because no difference between the two groups was observed in the cue condition (day 3) we reason that the deficit in in *+/Ex1a* mice is restricted to context-dependent mechanism, which has been previously associated with REM/fear memory consolidation [20], [21].

**Figure 5:**
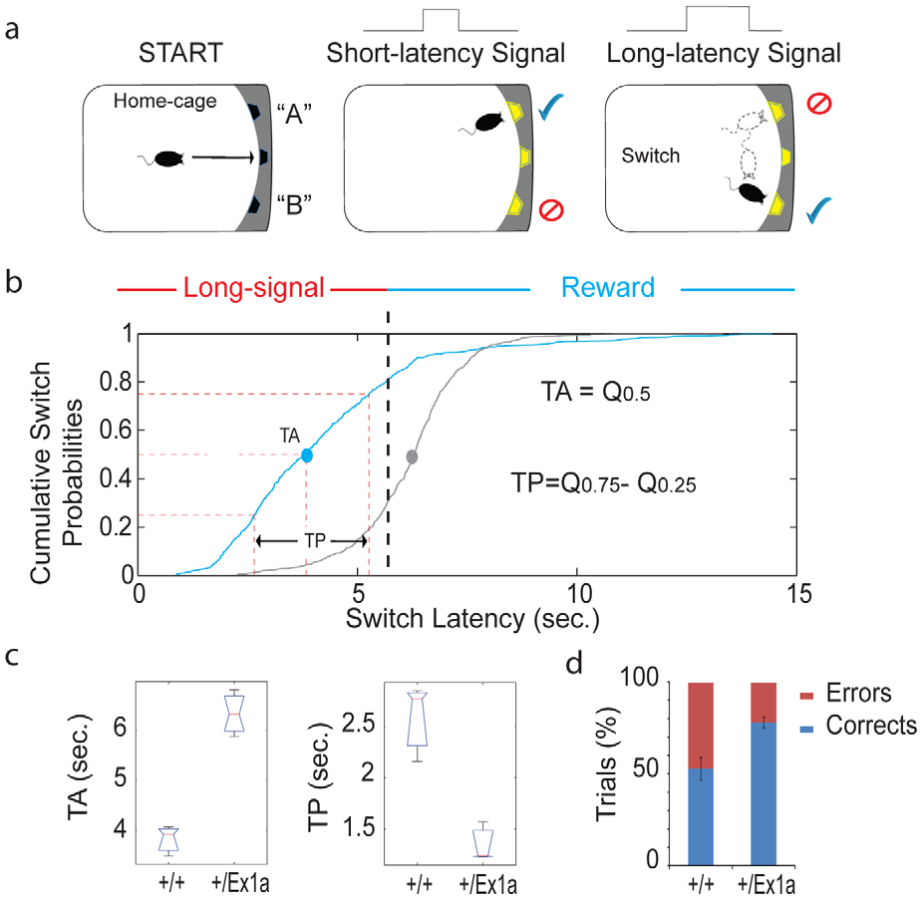
Cognitive performance in Exon 1A mice and littermate controls. (a) A schematic representation of the “switch task” in which a mouse self-initiates the trial (“START”) and receives a reward according to the duration of the signal (“short-latency” versus “long-latency”) and the location (“A” or “B”). (b) The cumulative distribution of all switch latencies (the time at which the mouse stops nose poking in the short-location and moves to the long location) for all long-signal trials are reported for the +/Ex1a mice and their littermate controls. (c) From the above cumulative distribution of the switch latencies we have derived the Time Accuracy (TA), as the median (Q_0.5_), and the Time Precision (TP), as the difference of the third and first inter-quartiles (Q_0.75_−Q_0.25_), similar to what is described in [22]. Box-plots of TAs (the median values for each subject) and TPs (the inter-quartiles) are shown as well as (d) the percentage of correct versus error trials for the whole experiment. The correct/error trials graph refers only to the “Switch” condition, however, the same trend was observed in the “Probes” condition.

A different subset of mice was tested in their home-cage with a cortico-striatal cognitive task, the “Switch-test” [22]. The test requires the animal to decide to nose-poke in different hoppers within the home-cage in response to a short- versus a long-light signal to obtain a reward (Figure 5a). Optimal performance in this task implies that the animal has learnt to distinguish between a short-signal associated with the reward coming at the hopper in one of the two locations and a long-signal associated with the reward coming at the hopper in the other location. This test of cognitive performance assesses whether the animal has an accurate representation of both endogenous/implicit (the subjective estimation of the signal duration) and exogenous/explicit (the ratio between short and long signals) temporal variables. In one condition (the “Switch” condition) all trials resulted in a reward if the animal responded correctly. In a second condition (the “Probes” condition) a percentage of trials was never rewarded regardless of the response of the animal. This latter condition was to measure the animal's uncertainty and its cognitive performance during a temporal decision making process [23]. Interestingly, *+/Ex1a* mice performed better in each phase of the experiment showing higher accuracy and time precision compared to controls (Figure 5b–5d). As expected, this 24-hour home-cage cognitive effort triggered a significant NREM increase and a higher delta power in *+/Ex1a* mice compared to *+/+* mice in both “Switch” and “Probes” conditions (Figure S1a–S1c). Notably, the “Probes” condition, which added a degree of uncertainty to the expectation of obtaining a reward, resulted in an more severe augmentation of NREM sleep respect to the “Switch” condition. REM did not change significantly following both conditions neither in *+/+* or *+/Ex1a* mice (Figure S1a–S1c).

## Discussion

We have shown here clear evidence for an involvement of the imprinted *Gnas* transcript in the modulation of sleep and sleep-dependent behaviors.

Our study demonstrated a close link between sleep and thermoregulation. Temperature control is a well-known mechanism that modulates the expression of REM/NREM sleep in humans and mice [24]. When the thermoregulatory demand increases, REM sleep diminishes in rodents [24]–[26]. Hence, the increase of body temperature in *+/Ex1a* accounts for the significant reduction of REM sleep that we have observed in these mutants. Moreover, several studies have shown that an increase in body temperature is associated with an increase in NREM sleep propensity [24], [27]–[29]. Thus, the hyperthermic phenotype is responsible for the REM defect-NREM improvement that we observed in *+/Ex1a* mice, indicating a link between imprinted *Gnas*, thermoregulation and REM/NREM sleep [24]. The molecular pathway joining *Gnas* and thermoregulation in BAT is well understood. G_s_α is considered an important mediator of the activity of the sympathetic nervous system (SNS) on many functions of BAT, included thermogenesis and energy expenditure [30]. SNS stimulation of BAT leads to thermogenesis. G_s_α is a constituent of transmembrane G-protein-coupled receptors that translate adrenergic SNS stimulation, in BAT, to the activation of UCP1 via specific intracellular changes and a signaling cascade that involves the production of cAMP and activation of protein kinase A (PKA). Our results showing increased *Gnas* - *Ucp1* expression are in line with a specific SNS-molecularly mediated cAMP-PKA role in non-shivering thermogenesis and therefore sleep. Indeed, the intracellular activity of cAMP-PKA is inversely related to the urge to sleep. In particular, an increased PKA affects sleep according to the specific cells in which it is expressed [31]–[33]. A reduced REM sleep would, presumably, be associated with an elevated cAMP-PKA activity.

We have shown that functional monoallelic expression of *Gnas*, due to imprinting of this gene, is important, in mice, for the physiology of REM sleep and for the consolidation of fear conditioning contextual memories. Many experimental observations support the idea that hippocampus is crucial for the development of contextual fear conditioning [20], [34]. However, fear conditioning responses are mediated by a complex interaction within limbic and prelimbic areas and this is also modulated by circadian genes [35]. As Chen and colleagues [36], have shown that *Gnas* is not imprinted in the hippocampus, our study implies that this specific cognitive phenotype must be due to imprinting of *Gnas* in brain regions other than hippocampus. Within this same study it was shown that *Gnas* is imprinted in the paraventricular nucleus (PVN) of the hypothalamus, a brain region that subserves important metabolic functions [36]. The PVN receives important projections from neurons located within the suprachiasmatic nucleus (SCN) of the hypothalamus, the master clock for circadian rhythms [37]. The interaction between PVN and SCN is important in orchestrating circadian rhythms and to set proper neuroendocrine responses to stressors [37]. Thus loss of *Gnas* imprinting, within these structures, can be envisaged as playing a role in both sleep and behavior. The results of our study confirm the idea that REM sleep is fundamental in the consolidation of fear responses [19], likely, involving a complex network of brain activity.

We have also shown that NREM functions are sensitive to *Gnas* dosage but, in this case, loss of imprinting of *Gnas* results in a reduction of uncertainty in temporal decision making. This improvement is paralleled by an increased contribution of slow wave activity in NREM sleep. Our result is consistent with the idea that cortical slow oscillations, by modulating synaptic circuits between subcortical and cortical structures, are responsible for the consolidation of daily memories during sleep [13].

In conclusion, in our study, loss of imprinting of *Gnas*, inhibits REM and primitive REM-linked functions, such as the fear response to a threatening context. Conversely, it enhances NREM physiology and high-level cognitive functions that developed alongside a progressively complex brain.

However, from a behavioral point of view an increased precision in interval timing estimation does not necessarily result in a better performance in other behavioral responses. Indeed, if the subject is less certain about the time of the foot-shock, its fear conditioning response may start earlier and stop later [38], hence resulting in a higher freezing time. Perhaps, evolution has developed a balanced mechanism between temporal uncertainty and fear behavioral responses and then loss of imprinting, in *+/Ex1a* mice, involves a reduction of freezing because of a higher timing precision. Thus, REM/NREM sleep expression may favor this well-adjusted mechanism.

The results of our study indicate a specific role for the imprinted gene *Gnas* in thermoregulation, which in turn affects REM/NREM sleep and then, cognitive performance. In addition we also reported a novel effect of specific cognitive mechanisms on sleep, by showing that a specific decision making process, the consolidation of an interval timing task, influences NREM sleep. Furthermore, in our mouse mutant model, the particular NREM physiology, exacerbates the effect of cognition on sleep homeostasis. This study attests the relevance of *Gnas* in brain functions and that loss of imprinting of *Gnas* affects cognitive processes.

Another transcript within the *Gnas* locus, the paternally expressed transcript *Gnasxl*, is expressed in specific sleep-related brain areas including the locus ceruleus and cholinergic laterodorsal tegmental nuclei [39]. The activity of neurons in the cholinergic laterodorsal tegmental nucleus is particularly important in the regulation of REM sleep [40], [41]. Mice with mutations in paternally derived *Gnasxl* transcripts show phenotypic deficits associated with growth and development, which is a critical stage for REM sleep across many species [4]. Thus *Gnasxl* may also play a role in sleep.

## Methods

### Mouse breeding, genotyping, and procedures

Initial *Ex1a* stock mice were produced in MRC-Harwell on 129/SvEv background and then transferred to IIT. In IIT mice were bred and maintained, through paternal inheritance, for several generations on C57BL/6J background, as this is a favorite background for behavioral studies. The genotyping of the mice was conducted following the assay as indicated in [42]: Exon1aF 5′cagtcgcgtcggcaccgcggag3′ and Exon1aR 5′gacgcactcacacgcaaagcag3′.

All the behavioral and electrophysiological experiments, in adult *+/Ex1a* mice and *+/+* littermate controls, were conducted in the home-cage environment. In addition, mRNA expression profiles for *Gnas* and Uncoupling Protein (*Ucp*)1 were made in naïve adult mice. Each experiment included 8 male mice (10-weeks old) for each genotype and all procedures were done under the guidance issued by the UK authority (Project Licence Numbers 30/2526) and under the Italian Policy (licence issued on 19/06/2009, decreto N°106/2009-B).

### Quantitative real-time PCR

Total RNA was extracted from about 0.2 g of snap frozen brown adipose tissue (BAT) and homogenized using Pestel with Trizol Reagent (Invitrogen, Carlsbad, CA) to isolate total RNA according to the manufacturer's procedure. Q-PCR was conducted essentially as previously described [43], [44]. Specific primers were: UCP1:f5′-GTCCCCTGCCATTTACTGTCAG-3′, r5′-TTTATTCGTGGTCTCCCAGCATAG-3′; *GNAS*: f5′-AGAAGGACAAGCAGGTCTACCG-3′, r5′-GTTAAACCCATTAACATGCAGGA-3′; β-actin, f5′-GGCACCACACCTTCTACAATG-3′, r5′-GGGGTGTTGAAGGTCTCAAAC-3′. Thermal cycling parameters were: denaturation 95°C for 5 min followed by 40 cycles of denaturing- annealing and extending (95°C for 15 sec, 60°C for 30 sec and then 70°C for 1 min). The results were calculated by the comparative *C*_t_ method according to the Applied Biosystems ABI-PRISM-7700 User Bulletin#2. Each sample was run in quadruplicate to obtain average *C*_t_ values and a Δ*C*_t_ value for the target gene of the same sample, normalizing each sample to β-actin. The expression relative to β-actin was determined by calculating 2^−Δ*C*t^. Mean comparison was performed with unpaired Student's t-test.

### Behavioral experiment

Mice were subjected to a long-term investigation in home-cage environment after the implant of a wireless system (Data Sciences) that enables to record electroencephalography (EEG), electromyography (EMG), locomotor activity and body temperature for off-line sleep-stage analysis (see [19]). Automated sleep scoring followed by visual manual inspection was performed using all sleep criteria for mice [19]. We performed Fast Fourier Transform analysis of the EEG signals with SleepSign software. The contributions of EEG frequencies was expressed as power densities in each frequency bin in all NREM and REM sleep epochs (as described in [45]).

A 2 week post-surgery period of recovery were given to each mouse to ensure a full recovery of normal sleep. At the end of the recovery period, we started recording all the physiological signals uninterruptedly for 48 consecutive hours (sleep baseline). Then, mice went under 6-hour sleep deprivation (SD) and 6-hour recovery period. After an additional 1-week the mice underwent fear conditioning (FC) or timing learning (Switch task) conditions.

#### The “Switch task.”

Mice were maintained in home-cage (equipped with a novel three-hopper operant wall from TSE) to perform in the switch task [22], The test requires the animal to decide to nose-poke in response to a light-signal to obtain a reward (Figure 5a). All trials were self-initiated by the animals by nose poking in one hopper (located in the middle of the operant wall) in the home-cage. The rewards were delivered through the two lateral hoppers; one hopper was always associated with short light-signals while the other hopper was always associated with long light-signals. On a fraction of the trials, the reward pellets were available in location “A” after a short-signal duration (3-seconds). On the other trials, the pellets were available after a long-signal duration (6-seconds) at the location “B”. If and when they reckoned that the short-latency had elapsed, they decided to leave the location “A” and move (switch) to the location “B”. The rewards were obtained only if the first nose poke of the animal occurred at the correct location. If mice switched too soon or too late for those long-signal trials, they ended up without payoff.

We trained all mice to a steady-state performance, keeping 1∶3 ratio between short- and long- signals (3 vs. 9 sec respectively). Then we reduced the short-/long-signal ratio to 1∶2 (3 vs. 6 sec), which is effectively similar to increased endogenous timing uncertainty (i.e. [23]). The test included two experimental conditions: “Switch” and “Probes”. The “Probes” condition differs from the “Switch” condition as we introduced 20% of no-reward(probe)-trials to independently test the timing uncertainty. We analysed for each condition the number of errors as well as switch latencies, time accuracy and time precision (as described in [22], [38]).

#### Fear conditioning

Eight mice for each genotype were tested while on their active/dark phase. On day 1 each mouse was placed in a fear conditioning chamber and, after a 2-min baseline, were given four light/white-noise (55-dB) cue-stimuli lasting 30 s and overlapping in the last 2 s with a footshock, (0.5 mA) lasting further 2 s. Each pairing was 2 min apart (inter-trial interval). After a final 30 s post-footshock delay, mice were returned to their home cages. To test contextual memory, we placed the mice back in the original chamber (24-hr post-training, day 2) for 3 minutes with no footshock during which freezing bouts were scored. During day 3 we placed all mice, for 3 minutes, in the testing chamber but with a different context respect to the original one. During this exposure, we subjected all the animals to the light/white-noise cues of day 1 and we measured their freezing behavior.

## Supporting Information

Figure S1 Multiple-rebound values are plotted for NREM (a) and REM (b) after sleep deprivation (SD), FC and during the “Switch” and “Probes” condition of the Switch-task. The interrupted circular lines indicate the 100% baseline values. (c) Mean power densities of frequencies in NREM (red lines) and REM (blue lines) epochs are reported for all experimental conditions.
(PDF)

Figure S2 Four-hour bins percentage and shadowed ± s.e.m of REM (lower panel) sleep during 6-hour sleep deprivation (SD) and the following 6-hour recovery period. Grand-averages and shadowed ± s.e.m. of body temperature (upper panel) are plotted for the same period.
(PDF)
