# Supplementary figures and images for: Loss of Gnas Imprinting Differentially Affects REM/NREM Sleep and Cognition in Mice
Source: PLoS Genet. 2012 May 10;8(5):e1002706. doi: 10.1371/journal.pgen.1002706 (PMC3349741; doi:10.1371/journal.pgen.1002706)

a

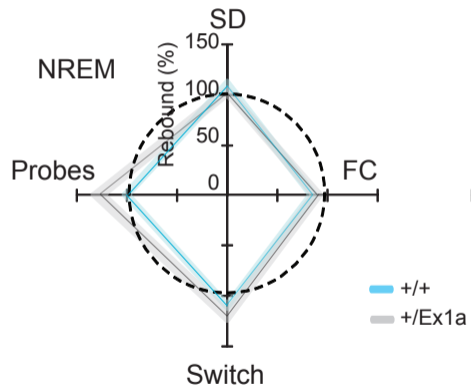

b

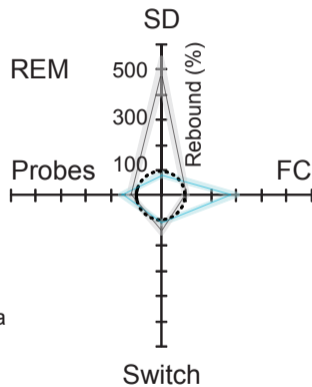

c

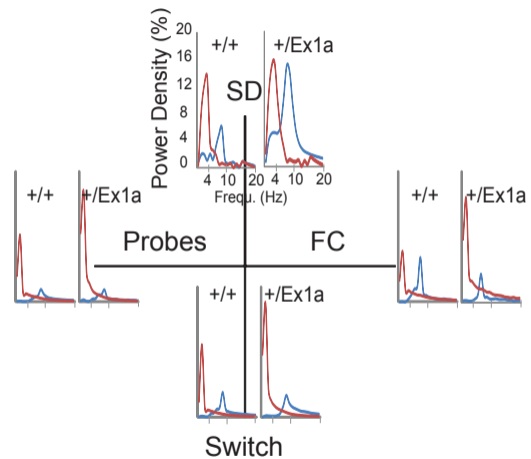

Supplement: Figure S1 — Multiple-rebound values are plotted for NREM (a) and REM (b) after sleep deprivation (SD), FC and during the “Switch” and “Probes” condition of the Switch-task. The interrupted circular lines indicate the 100% baseline values. (c) Mean power densities of frequencies in NREM (red lines) and REM (blue lines) epochs are reported for all experimental conditions. (PDF) [file pgen.1002706.s001.pdf]

Lassi et al. Figure S2

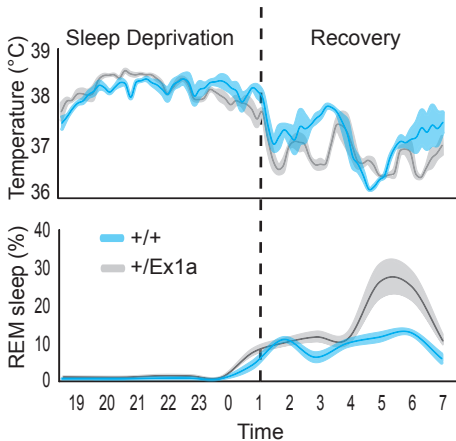

Supplement: Figure S2 — Four-hour bins percentage and shadowed ± s.e.m of REM (lower panel) sleep during 6-hour sleep deprivation (SD) and the following 6-hour recovery period. Grand-averages and shadowed ± s.e.m. of body temperature (upper panel) are plotted for the same period. (PDF) [file pgen.1002706.s002.pdf]
